# Face Masks

**Published:** 1919-08

**Authors:** George A. Comte

**Affiliations:** Los Angeles, Cal.


### FACE MASKS

BY GEORGE A. COMTE, D.D.S., LOS ANGELES, CAL.

Now that the conflict is over, the interest of the world is centered on reconstruction, and the rehabilitation, both mental and physical, of those who have been maimed and disfigured in the war.

Due to conditions of trench warfare, a very large percentage of blesses are those who have received wounds in the head and upper portions of the body.

Of these the facial disfigurements are the most unsightly, and are at times repulsive to a degree; under these circumstances, a man's mental attitude will suffer, moroseness will develop, and despair will take possession of his being—in the language of the hour, his ''morale'' will be destroyed.

Wonderful achievements have been obtained by plastic surgery as practiced in Europe today, but results can only be obtained when sufficient tissue is at hand for proper manipulation. It was manifest that some provision was mandatory to restore these mutiles to usefulness, and it was with this idea in mind that I undertook a series of experiments to determine the material most suitable for the work in hand.

After thoroughly canvassing the ground and rejecting a number of materials that at first seemed satisfactory, a solution of celluloid was finally determined upon as being the most advantageous for the purpose.

The following is a brief description of the technic followed. If possible, a photograph is obtained of the patient, taken previous to the injury, in order that the parts may be built up along normal lines.

The depressions caused by the wound are filled out with wax, modeling compound, or plasticene, as the occasion requires; it sometimes being necessary to first bridge the cavities with cotton and adhesive tape.

Now the facial contour is built up to its proper conformation; this is perhaps the most difficult and tedious part of the work, and requires the maximum of artistic skill, yet when painstakingly done the results obtained fully compensate the operator for his labor.

When all is in readiness the eye-brows and other hairy surfaces are covered with oiled tissue paper or gold beaters skin, the whole surface given a coating of heavy petrolatum and an impression taken. Care must be exerted to place quills in the nostrils in such a manner as not to impede the patient's breathing. After removing the impression, it is coated with any good separating medium, and a cast is made, which when hardened sufficiently is separated in the usual manner, and any minor deficiencies corrected. The model is now thoroughly soaped and a smooth coating of thin tinfoil applied. Celluloid blanks, such as are used for platework, are dissolved in acetone or pyro-acetique (fr.) to the consistency of thick cream and applied to the model with a flat camel's hair brush. This is allowed to dry for three hours, when a second application is made. At this stage any irregularities are noted, and removed either by filling up air bubbles with a thickened solution of the celluloid, or by smoothing out rough places with wet pumice and cotton. As the pigmentation varies greatly in individuals, it is obvious that many different colored solutions must be used in successive layers; those most favored are white, clear, brown, pink, yellow, and a touch of green. The solutions are tinted with ordinary oil paints. By incorporating the pigment in the material of the mask itself, a distinct advantage is gained, as there is a certain amount of translucency not obtained in a painted mask. A final application of pumice is applied and the mask surface is well rubbed to lessen the gloss, when it is carefully lifted from the model, and the tinfoil removed by the application of metallic mercury, the surplus being wiped off with cotton. Spectacles are added in order to hold the appliance in place, and a mustache may be used to soften the lines of demarkation.

When carefully made these masks are undetectable at a distance of a few feet and self-confidence is restored to the point where the patient is enabled to take up the thread of life once more, and lead a life of usefulness among his fellow men.—*Pacific Gazette.*

